# Anti-Diabetic and Hepato-Renal Protective Effects of Ziyuglycoside II Methyl Ester in Type 2 Diabetic Mice

**DOI:** 10.3390/nu7075232

**Published:** 2015-07-07

**Authors:** Dong Ju Son, Seock Yeon Hwang, Myung-Hyun Kim, Un Kyu Park, Byoung Soo Kim

**Affiliations:** 1 College of Pharmacy & Medical Research Center, Chungbuk National University, Cheongju, Chungbuk 361-951, Korea; E-Mail: sondj1@hotmail.com; 2 Department of Biomedical Laboratory Science, College of Natural Science, Daejeon University, Daejeon 300-716, Korea; E-Mails: syhwang@dju.kr (S.Y.H.); saladin04@naver.com (U.K.P.); 3 Department of Physiology, College of Korean Medicine, Daejeon University, Daejeon 300-716, Korea; E-Mail: abeliophylum@naver.com

**Keywords:** ziyuglycoside, *Sanguisorba officinalis*, diabetes, diabetic hepatopathy, diabetic nephropathy

## Abstract

Type 2 diabetes is a metabolic disorder caused by abnormal carbohydrate metabolism, and closely associated with abnormal lipid metabolism and hepato-renal dysfunction. This study investigated the anti-diabetic and hepato-renal protective properties of ziyuglycoside I (ZG01) derivative on type 2 diabetes. ZG01 was isolated from roots of *Sanguisorba officinalis* and chemically modified by deglycosylation and esterification to obtained ziyuglycoside II methyl ester (ZG02-ME). Here, we showed that ZG02-ME has stronger anti-diabetic activity than the original compound (ZG01) through decreasing blood glucose, glycated hemoglobin (HbA1c), and insulin levels in a mouse model of type 2 diabetes (*db*/*db* mice). We further found that ZG02-ME treatment effectively ameliorated serum insulin, leptin and *C*-peptide levels, which are key metabolic hormones, in *db*/*db* mice. In addition, we showed that elevated basal blood lipid levels were decreased by ZG02-ME treatment in *db*/*db* mice. Furthermore, treatment of ZG02-ME significantly decreased serum AST, ALT, BUN, creatinine, and liver lipid peroxidation in *db*/*db* mice. These results demonstrated that compared to ZG01, chemically modified ZG02-ME possess improved anti-diabetic properties, and has hepato-renal protective activities in type 2 diabetes.

## 1. Introduction

Diabetes mellitus is a group of metabolic disorders caused by an abnormal carbohydrate metabolism and mainly linked to abnormal blood glucose and insulin levels or an ineffective response of cells to insulin [1]. The International Diabetes Federation predicts that by 2035 10% of the population of the world will have been diagnosed with diabetes, raising serious concerns over the resulting elevated morbidity and mortality as well as the impact on health care budgets [2]. Type 2 diabetes mellitus, the most common type of diabetes, it is a lifestyle-related diabetes or non-insulin-dependent diabetes and is a fast-growing and potentially life-threatening disease [3]. In clinical therapy for type 2 diabetes, hypoglycemic agents such as metformin, α-glycosidase inhibitors, thiazolidines, and sulfornylurea derivatives are available for use. However, these compounds have side effects including weight-gain and cardiovascular problems, and providing them at a relatively low cost is still a challenge to the medical system [4]. Having comparatively low side effects and low cost, phytochemicals from natural resources are receiving significant attention for therapeutic potential as anti-diabetic agents and as appropriate treatment modalities for the safe treatment of various metabolic diseases including diabetes [5,6,7,8,9,10].

The dried root of *Sanguisorba officinalis* L. (Rosaceae), also known as “Ziyu”, has been used as a traditional medicinal plant in East Asia, and is used primarily to treat hemostasis and inflammation [11]. It is well documented that *S. officinalis* possesses multiple pharmacological activities including antioxidant [11,12], anti-tumor [13,14,15], anti-infection [16,17] and immunomodulatory [18,19] actions. A variety of chemical constituents were isolated from *S. officinalis* and their biological properties were evaluated [13,20,21]. Ziyuglycosides are a group of primary terpenoid constituents in *S. officinalis* that has been shown to exhibit a wide ranges of biological actions, including hemostasis [22] anti-cancer [23,24,25], anti-inflammation, and anti-skin aging [26,27]. Ziyuglycoside II (ZG02) is a triterpenoid compound isolated from *S. officinalis* [28]. Previous studies demonstrated that the methyl ester form of ZG02 (ZG02-ME) plays a significant role in modulating hyperlipidemic conditions [29,30,31] and inflammatory responses [32]. Furthermore, it has been reported that ZG02-ME, which is derivatized from ziyuglycoside I (ZG01) by chemical modification, possesses an improved inhibitory activity *in vivo* compared with that of ZG01 on tissue factor and tumor necrosis factor (TNF)-α productions [33]. However, no previous studies have evaluated anti-diabetic activity of ziyuglycosides in an *in vivo* setting, especially on the non-insulin-dependent diabetes (Type 2 diabetes). Accordingly, the present study was performed to investigate the role of ziyuglycosides in regulating blood glucose, plasma insulin, and serum lipid parameter levels in *db*/*db* mice, a mouse model of diabetes type 2, along with the renal and liver function parameters.

## 2. Experimental Section

### 2.1. Plant Material and Preparation of ZG02-ME

**Figure 1:**
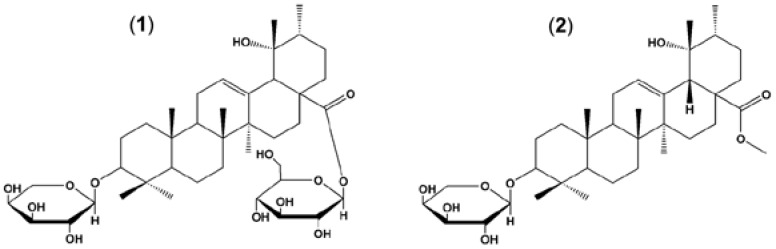
Structure of ziyuglycoside I and ziyuglycoside II methyl ester. (**1**) ziyuglycoside I (ZG01); (**2**) ziyuglycoside II methyl ester (ZG02-ME).

The roots of *Sanguisorba officinalis* were purchased from an herbal materials supply house (Youngcheon, Kyungpook, Korea) and identified at the Department of Plant Resources, Hankyung National University (Ansung, Gyeonggi, Korea) by Professor Tae-Wan Kim. A voucher specimen was deposited at Department of Plant Resources, Hankyung National University (HKU2012-142). The air-dried roots of *S. officinalis* (3 kg) were powdered and exhaustively extracted with 95% methanol (MeOH) (3 × 9.0 L) at room temperature for 3 days. The extract was filtered and concentrated under reduced pressure on a rotatory evaporator at 45 °C, resulting in 448.0 g of crude MeOH extract. The entire MeOH crude extract was defatted with cy-clohexane, and subjected to column chromatography using Diaion resin HP-20 with a stepwise gradient of MeOH containing increasing amount of H_2_O (from 100% down to 0%). On the basis of similar thin layer chromatography (TLC) profiles, four pooled fractions (fr); fr.SO-1 (208.00 g), fr.SO-2 (56.52 g), fr.SO-3 (106.14 g) and fr.SO-4 (66.50 g) were obtained. Purification of fr.SO-3 with repeated Diaion resin HP-20 chromatography using a stepwise gradient of MeOH containing increasing amount of H_2_O (from 70% down to 0%) provided seven sub-fractions (sfr.); sfr.SO-3-1 (3.73 g), sfr.SO-3-2 (28.07 g), sfr.SO-3-3 (12.21 g), sfr.SO-3-4 (10.92 g), sfr.SO-3-5 (30.70 g), sfr.SO-3-6 (12.35 g) and sfr.SO-3-7 (8.14 g). The sfr.SO-3-6 was purified using HPLC (Capcell Pak C18 UG120 5 µm, 4.6 × 150 mm, Shiseido Co., Japan) with a gradient of acetonitrile containing increasing amount of H_2_O using the following gradient program; 0 min (20%), 0–20 min (40%), 40–60 min (80%), 60–90 min (100%) after column chromatography over silica gel (230–400 mesh) with a MeOH–H_2_O gradient (20%–100%). This process yielded 1.5 g of final purified compound which was identified as (**1**) ZG01 by ^1^H-NMR and ^13^C-NMR, the results of which were in agreement with previously published data [34]. To obtain (**2**) ZG02-ME, (**1**) ZG01 was chemically modified using previously described methods [33]. Briefly, purified (**1**) ZG01 (1 g) dissolved in normal butanol (*n*-BuOH) (20 mL), H_2_O (8 mL) and sodium hydroxide (NaOH) (1.88 g), and refluxed for 5–6 h for the deglycosidation reaction. After neutralizing, the reaction product was partitioned with chloroform (CHCl_3_), and the CHCl_3_ layer was continuously washed with saturated sodium bicarbonate (NaHCO_3_) and saturated sodium chloride (NaCl). The fraction with ZG02 was obtained after dehydration with magnesium sulfate (MgSO_4_), then ZG02 (670 mg, yield = 67%) was isolated by column chromatography. Purified ZG02 (500 mg) was dissolved in dimethylformamide (5 mL), NaOH (50 mg) and MeOH (0.1 mL). The reaction mixture was treated with methyl iodide (0.15 mL) and then shaken for 24 h at room temperature for esterification. The reaction product was partitioned with ethyl acetate (EtOAc), and then EtOAc layer was mixed with saturated NaHCO_3_, washed with saturated NaCl, dehydrated with MgSO_4_ filtered to obtain ZG02-ME fraction. The obtained ZG02-ME fraction was eluted by column chromatography to prepare pure (**2**) ZG02-ME compound (475 mg, yield = 95%). The chemical structure of (**2**) ZG02-ME was determined (Figure 1) by ^1^H-NMR, the result of which was in agreement with previously reported data [33].

### 2.2. Animals and Administration

All animal procedures were approved by the Animal Care and Use Committees at Daejeon University. Male C57BLKS/J lar +Lepr^db^/+Lepr^db^ (*db*/*db*) diabetic mice (10 weeks old upon receipt) were purchased from Japan SLC, Inc. (Hamamatsu, Japan), and were fed *ad libitum* with standard chow diet until treatment at 12 weeks of age in a constant environment (room temperature 23 ± 1 °C, room humidity 50%–60%) with a 12 h light/dark cycle. ZG01 and ZG02-ME were dissolved in normal saline containing 0.01% DMSO and then sonicated for 10 min. The *db*/*db* mice were intragastrically administrated once a day for 4 weeks with normal saline (vehicle, *n* = 8), ZG01 (5 mg/kg, *n* = 8) or ZG02-ME (1 to 5 mg/kg, *n* = 8/group), respectively, at a range of doses which was suggested in previous report [33]. Age-matched C57BLKS/J Iar m+/+Lepr^db^ (*db*/*m*) mice were selected as the normal control group. At the end of the intervention, all mice were fasted overnight and then sacrificed. Fasting blood was collected, and the liver samples were collected. Liver tissues and sera were stored at −80 °C until further analysis.

### 2.3. Body Weight and Food Consumption Measurements

Changes of body weight were measured daily for 4 weeks, from one day before initiation of administration using an automatic electronic balance (Precisa Instrument, Zurich, Switzerland). For food and water consumption measurements, all mice were allocated in individual cages containing 100 g of food and 250 mL of water. Unconsumed food and water were measured at 24 h after it had been supplied using an automatic electronic balance or a measuring cylinder, respectively. These are regarded as individual daily food (g/24 h/mouse) and water (mL/24 h/mouse) consumption of mice.

### 2.4. Blood Biochemistry and Lipid Peroxidation

At the end of the treatment, all mice were fasted overnight and then euthanized. Blood was collected from vena cava, and collected, then centrifuged at 15,000 rpm for 10 min at room temperature for separating serum. Blood glucose, serum triglycerides (TG), serum total cholesterol (TC), low-density lipoprotein cholesterol (LDL-C) and high-density lipoprotein cholesterol (HDL-C), aminotransferase (AST), alanine aminotransferase (ALT), blood urea nitrogen (BUN) and creatinine were measured using Hitachi-7080 auto analyzer (Hitachi Ltd., Tokyo, Japan) using diagnostic kits (Wako Diagnostic, Richmond, VA, USA). The whole blood glycosylated hemoglobin A1c (HbA1c), an indicator of plasma glucose concentration over prolonged periods [35], concentration was measured with an automated hemoglobin testing system (Variant-II, Bio-Rad Laboratories Inc., Hercules, CA, USA). The level of lipid peroxidation in liver and kidney tissues were determined as previously described [36]. Briefly, separated liver and kidney tissues were homogenized in ice-cold Tris-HCl (pH 7.4), then liver lipid peroxidation was determined by estimating malondialdehyde (MDA) using the thiobarbituric acid (TBARS) test at absorbance 525 nm, as nmole of MDA/mg protein.

### 2.5. Measurement of Serum Insulin, C-Peptide, and Leptin

Serum insulin (Diagnostics Products Co., Los Angeles, CA, USA), *C*-peptide (Double-antibody RIA, Diagnostics Products Co.) and serum leptin (Linco Research Inc., St Charles, MO, USA) levels were detected using a commercially available radioimmunoassay (RIA) kits according to the manufacturer’s instructions.

### 2.6. Statistical Analysis

Statistical analyses were carried out with Graph-Pad Prism 5.0 (GraphPad Software Inc., La Jolla, CA, USA). Pairwise comparisons were performed using one-way Student’s *t*-tests. Data are presented as means ± standard deviation (SD) of the indicated number of experiments. Differences between groups were considered significant at *p*-values below 0.05.

## 3. Results

### 3.1. Effects of Ziyuglycosides on Blood Glucose and Insulin Levels in db/db Mice

Initially, we carried out a functional activity assay to evaluate the anti-diabetic activity of ziyuglycosides on type 2 diabetes using *db*/*db* mice. The *db*/*db* mice were treated with a single dose of ZG01 or ZG02-ME (5 mg/kg body weight/day) for one week. The results are shown in Table 1. As contrasted with normal control group (saline-administrated *db*/*m* mice), *db*/*db* mice showed significantly higher blood glucose levels that were lowered by (**1**) ZG01 and (**2**) ZG02-ME treatment with reduction rates of 2.6% and 11.4%, respectively. We further observed that the glycated hemoglobin (HbA1c) concentration was higher in *db*/*db* mice, which was significantly reduced by treatment with ZG01 and ZG02-ME with reduction rates of 19.4% and 37.3%, respectively. In addition, we observed that the serum insulin level was also significantly decreased by ZG02 and ZG02-ME treatment in *db*/*db* mice. These results indicated that ZG02-ME has stronger blood glucose and serum insulin level down-regulating activity in diabetic-obese *db*/*db* mice than that of ZG01.

Since, ZG02-ME was identified to have anti-diabetic property on the type 2 diabetes, we have further evaluated its anti-diabetic effect with consecutive doses on a four weeks treatment setting. After four weeks treatment, we found that ZG02-ME treatment at doses of 1, 3, and 5 mg/kg body weight daily significantly reduced the blood glucose level in *db*/*db* mice compared to vehicle control group in a dose-dependent manner with reduction rates of 11.1%, 17.6%, and 22.4%, respectively. We further confirmed that the HbA1c level was significantly reduced by treatment with ZG02-ME compared to vehicle-treated *db*/*db* mice which was consistent with results from initial experiments (Table 2). Taken together, these findings indicated that the administration of ziyuglycosides, especially ZG02-ME, have an anti-diabetic activity against type 2 diabetes.

**Table 1:** Effect of treatment of ziyuglycosides on glucose, HbAlc, and insulin levels in diabetic-obese (*db*/*db*) mice.

Treatment	Dose (mg/kg Body Weight)	Glucose (mg/dL)	HbA1c (%)	Insulin (ng/mL)
Normal control	–	112.3 ± 6.9	5.96 ± 0.04	2.42 ± 0.19
Vehicle control	–	693.5 ± 52.3 ^#^	8.16 ± 1.25 ^#^	3.67 ± 0.54 ^#^
ZG01	5	675.2 ± 86.7	6.58 ± 1.13 *	2.96 ± 0.49 *
ZG02-ME	5	614.4 ± 55.6 *	5.12 ± 1.32 *	2.74 ± 0.67 *

The *db*/*m* mice were intragastrically administrated daily with normal saline containing 0.01% DMSO, as the vehicle, (normal control). The *db*/*db* mice were intragastrically administrated daily with vehicle (vehicle control), ZG01, or ZG02-ME, respectively, for one week. The blood glucose, glycated hemoglobin (HbA1c), and serum insulin were measured as described in the methods. Data are shown as mean ± SD of (*n* = 8). **^#^** *p* < 0.05 *vs.* normal control, and * *p* < 0.05 *vs.* vehicle-treated control as determined by one-way Student’s *t*-test.

**Table 2:** Effect of ZG02-ME on blood glucose and HbAlc levels in diabetic-obese (*db*/*db*) mice.

Treatment	Dose (mg/kg Body Weight)	Glucose (mg/dL)	HbA1c (%)
Normal control	–	152.3 ± 7.6	6.72 ± 0.94
Vehicle control	–	621.6 ± 10.26 ^#^	9.23 ± 1.66 ^#^
ZG02-ME	1	552.5 ± 18.2 *	8.26 ± 1.27
	3	512.4 ± 13.5 *	7.32 ± 1.43 *
	5	482.3 ± 19.6 *	7.13 ± 0.86 *

The *db*/*m* mice were intragastrically administrated daily with normal saline containing 0.01% DMSO, as the vehicle, for four weeks (normal control). The *db/db* mice were intragastrically administrated daily with vehicle (vehicle control) or ZG02-ME, respectively, for four weeks. The blood glucose and glycated hemoglobin (HbA1c) were measured as described in the methods. Data are shown as mean ± SD of (*n* = 8). ^#^ *p* < 0.05 *vs.* normal control, and * *p* < 0.05 *vs.* vehicle-treated control as determined by one-way Student’s *t*-test.

### 3.2. Effect of ZG02-ME on Serum Insulin, C-Peptide and Leptin Levels in db/db Mice

To investigate whether the administration of ZG02-ME for four weeks is able to improve levels of insulin and metabolic parameters, we tested insulin, leptin, and *C*-peptide level changes in ZG02-ME-administrated *db*/*db* mice. After four weeks treatment, we found that serum insulin, leptin and *C*-peptide levels were significantly higher in the vehicle control *db*/*db* mice than in the normal control *db*/*m* mice. Treatment of ZG02-ME reversed the insulin, leptin and *C*-peptide levels in *db*/*db* mice as compared to vehicle control group (Table 3).

**Table 3:** Effect of ZG02-ME on serum insulin, leptin, and *C*-peptide levels in diabetic-obese (*db*/*db*) mice.

Treatment	Dose (mg/kg Body Weight)	Hormone Parameters (ng/mL)		
		Insulin	Leptin	*C*-peptide
Normal control	–	2.7 ± 0.2	28.6 ± 2.1	1.66 ± 0.23
Vehicle control	–	3.4 ± 0.4 ^#^	45.5 ± 2.6 ^#^	1.88 ± 0.25 ^#^
ZG02-ME	1	3.1 ± 0.5	42.6 ± 5.2	1.75 ± 0.18
	3	2.9 ± 0.5 *	38.4 ± 3.9 *	1.72 ± 0.26 *
	5	2.7 ± 0.6 *	35.4 ± 2.4 *	1.69 ± 0.16 *

The *db*/*m* mice were intragastrically administrated daily with normal saline containing 0.01% DMSO, as the vehicle, for 4 weeks (normal control). The db/db mice were intragastrically administrated daily with vehicle (vehicle control) or ZG02-ME, respectively, for four weeks. The serum insulin, leptin, and *C*-peptide levels were measured as described in the methods. Data are shown as mean ± SD of (*n* = 8). **^#^** *p* < 0.05 *vs.* normal control, and * *p* < 0.05 *vs.* vehicle-treated control as determined by one-way Student’s *t*-test.

**Figure 2:**
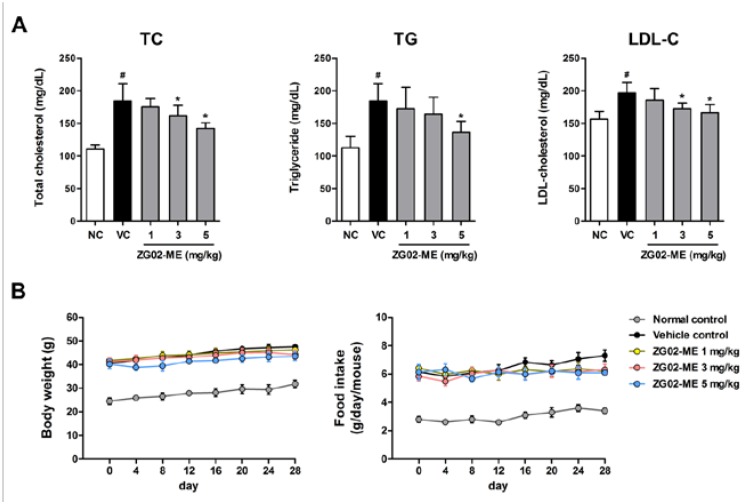
The effects of ZG02-ME administration on blood lipid levels, body weight gain, and food consumption in diabetic-obese (*db*/*db*) mice. The *db*/*m* mice were intragastrically administrated daily with normal saline containing 0.01% DMSO, as the vehicle, for four weeks (normal control). The *db*/*db* mice were intragastrically administrated daily with vehicle (vehicle control) or ZG02-ME (1, 3, 5 mg/kg body weight), respectively, for four weeks. (**A**) The total cholesterol (TC), triglyceride (TG), and low-density lipoprotein (LDL-C) levels in blood serum were measured as described in the methods; (**B**) Body weight and food intake were continuously measured from initial to final administration. Data are shown as mean ± SD of (*n* = 8). **^#^** *p* < 0.05 *vs.* normal control, and * *p* < 0.05 *vs.* vehicle-treated control as determined by one-way Student’s *t*-test.

### 3.3. Effect on the Basal Blood Lipid Levels in db/db Mice

We also investigated the effects of ZG02-ME administration on the blood lipid levels in *db*/*db* mice. Serum levels of TC, TG, and LDL-C were analyzed to determine whether ZG02-ME attenuates lipid metabolic abnormalities in *db*/*db* mice. We observed that the basal levels of TC, TG, and LDL-C in serum were significantly higher in *db*/*db* mice (vehicle control) than *db*/*m* mice (normal control). We found that ZG02-ME treatment for four weeks significantly reduced TC, TG and LDL-C levels in *db*/*db* mice compared with vehicle-treated control group (Figure 2A). We also observed that body weights and food intakes were greater in the *db*/*db* mice than in the *db*/*m* mice from the start of the experiments. We found that ZG02-ME treatment slightly deceased body weight gain and food consumption as compared with vehicle-treated *db*/*db* mice, but no significant differences between vehicle-control group and ZG02-ME-treated groups throughout the study period (Figure 2B).

**Figure 3:**
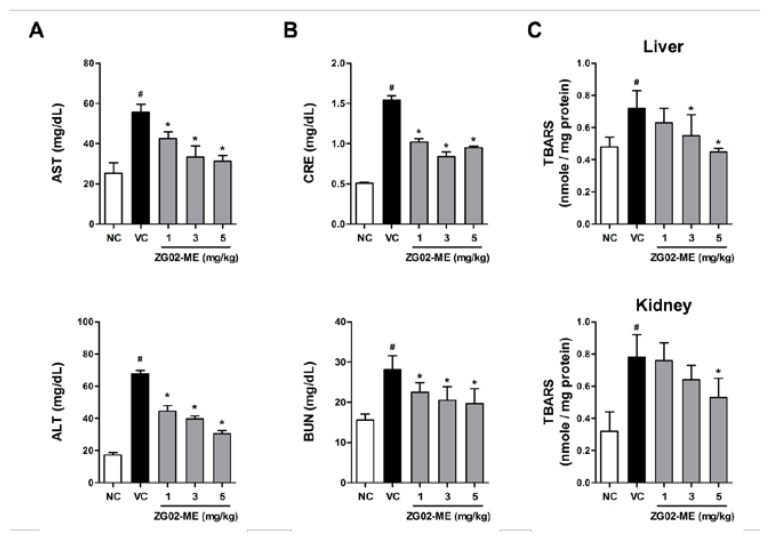
The effects of ZG02-ME treatment on AST, ALT, creatinine, BUN, and TBARS in diabetic-obese (*db*/*db*) mice. The *db*/*m* mice were intragastrically administrated daily with normal saline containing 0.01% DMSO, as the vehicle, for four weeks (normal control, NC). The *db*/*db* mice were intragastrically administrated daily with vehicle (vehicle control, VC) or ZG02-ME (1, 3, 5 mg/kg body weight), respectively, for four weeks. (**A**) Aspartate aminotransferase (AST) and alanine aminotransferase (ALT) levels as well as (**B**) creatinine (CRE) and blood urea nitrogen (BUN) levels in blood serum were measured; (**C**) Thiobarbituric acid reactive substances (TBARS) levels in liver and kidney tissues were also analyzed as described in the methods. Data are shown as mean ± SD of (*n* = 8). **^#^** *p* < 0.05 *vs.* normal control, and * *p* < 0.05 *vs.* vehicle-treated control as determined by one-way Student’s *t*-test.

### 3.4. Effect on Liver and Renal Function in db/db Mice

To investigate effect of ZG02-ME administration on liver function, we tested AST and ALT, liver function parameters, levels in blood serum. We observed that basal levels of AST and ALT in blood serum were significantly higher in *db*/*db* mice (vehicle control) than *db*/*m* mice (normal control). We found that ZG02-ME treatment for four weeks significantly reduced serum AST and ALT levels in *db*/*db* mice compared with vehicle control group (Figure 3A). We further found that higher levels of creatinine and BUN levels, kidney function parameters, in *db*/*db* mice compare with levels in the normal control group, which were significantly decreased in ZG02-ME-treated *db*/*db* mice. In addition, treatment of *db*/*db* mice with ZG02-ME showed decreasing activity against lipid peroxidation (TBARS values) both in liver and kidney tissues (Figure 3C). Together, these results demonstrated that the ZG02-ME treatment improves liver and renal functions in type 2 diabetic-obese mice.

## 4. Discussion

Type 2 diabetes is a metabolic disorder with various pathological manifestations and is closely associated with abnormal glucose and lipid metabolism. The worldwide prevalence of type 2 diabetes is steadilygrowing, and its related complications are serious health problems. Despite recent advancement in the management and treatment of diabetes led by success of pharmacological therapeutics, the lack of response to current pharmaceutical drugs in a proportion of patients, as well as therapy discontinuation due to drug-side effects, are still unsolved significant limitations. Therefore, new strategies that modulate diabetes by ameliorating insulin resistance and hyperlipidemia along with improving liver and renal functions are still urgently needed. In the present study, we evaluated the anti-diabetic properties of ZG02-ME, which is chemically modified from ZG01 of *S. officinalis*. Additionally, we investigated the anti-hyperlipidemia activity, and hepato-renal protective potentials of ZG02-ME in a mouse model of type 2 diabetes (*db*/*db* mice).

Ziyuglycosides are known as primary terpenoid compounds of *S. officinalis* that have been reported to exhibit diverse biological actions, including anti-cancer, anti-inflammation, anti-skin aging, anti-hyperlipidemia, and hemostasis properties [22,23,24,25,26,27,28,29,30,31,32]. However, anti-diabetic properties of ziyuglycosides have not been reported yet. Interestingly, *Cho* and colleagues [33] reported that chemical modification of ZG01, which originated from *S. officinalis* by deglycosylation and esterification improves inhibitory effects on blood coagulation and pro-inflammatory responses in rodents compared with that of ZG01 [33]. For this reason, we evaluated whether ZG02-ME, a methyl ester of ZG01, shows a stronger anti-diabetic activity than ZG01 in *db*/*db* mice. The *db*/*db* mouse model develops hyperphagic obesity and nonketotic diabetes similar to non-insulin-dependent diabetes mellitus in human [37]. Thus, the *db*/*db* mouse model has been well accepted as a useful resource to understand and treat type 2 diabetic conditions. In the present study, therefore, we used *db*/*db* mice to investigate anti-diabetic properties of ZG02-ME on type 2 diabetes (non-insulin-dependent diabetes) in an *in vivo* setting.

We investigated whether chemical modification potentiates anti-diabetic properties of ziyuglycoside on type 2 diabetes; our initial functional activity assay results showed that one week treatment of ZG02-ME, a deglycosylated and esterificated ZG01 derivative, more effectively decreased diabetic parameters such as blood glucose, glycated hemoglobin, and insulin levels than ZG01 treatment in *db*/*db* mice, indicating ZG02-ME has enhanced anti-diabetic activity *in vivo*. Based on the above results, to verify the four weeks treatment effect, we further evaluated the effect of ZG02-ME on metabolic hormone parameters. Leptin, an adipocytokine, is a protein that circulates in proportion with body fat mass and provides information regarding the nutritional status and subcutaneous fat mass to the neural center that regulates feeding behavior, appetite and energy expenditure [38]. In addition, leptin levels reflect the amount of stored fat and degree of energy imbalance in diabetes [39]. *C*-peptide is produced in equal amounts to insulin and is the best measure of endogenous insulin secretion in patients with diabetes [40]. We found that consecutive doses of ZG02-ME administration for four weeks dose-dependently and significantly reduced blood glucose and HbA1c level accompanied with amelioration of insulin, leptin, and *C*-peptide profiles in *db*/*db* mice. Together, these findings demonstrated that ZG02-ME can effectively regulates hyperglycemia, which should be controlled in diabetes, through amelioration of metabolic hormone parameters in type 2 diabetes.

Diabetes is often associated with dyslipidemia (hyperlipidemia), which is a well-recognized and modifiable risk factor of cardiovascular disease, thus, the management of diabetic dyslipidemia has been implicated as a key element in multifactorial approach to prevent cardiovascular disease in patients with type 2 diabetes [41]. Since the most critical issues associated dyslipidemia are increased serum LDL, TG, and TC levels [42], the efficacy of anti-hyperlipidemic activity is generally evaluated based on the decreases in serum LDL, TG, and TC levels. We found that oral gavage of ZG02-ME reduced blood lipid profile levels in *db*/*db* mice without affecting bodyweight and food consumption, suggesting ZG02-ME has anti-hyperlipidemic activity along with anti-hyperglycemic property.

Liver is a main metabolic organ for glucose; in the fed state, which is stimulated by insulin to take more glucose from the blood and synthesize glycogen, thus reducing the postprandial hyperglycemia. In the starved state, on the other hand, the liver generates more glucose from non-carbohydrate carbon substrates to maintain an adequate blood glucose level. Under diabetic conditions, however, glycogen synthesis is inhibited while gluconeogenesis is abnormally enhanced due to the inefficient utility of glucose [43]. Therefore, liver function monitoring and liver disease prevention are important aspects in management and treatment of diabetic patients [44]. Diabetic hepatopathy, an under-recognized liver damage, has frequently been observed in diabetic patients [45]. Elevated level of AST and ALT in serum is a most common indicator of skeletal muscle necrosis and hepatocellular necrosis, that are reflect liver dysfunction. As diabetes progresses, hypertrophic changes in the cytoplasm of hepatocytes due to lipid deposition were observed with the elevated serum AST and ALT levels. Our blood biochemical examination results showed that serum AST and ALT levels in ZG02-ME-treated *db*/*db* mice were significantly decreased compared to that of vehicle-treated group. Diabetic nephropathy is also a serious complication of diabetes mellitus, which is the leading cause of end-stage renal disease [46]. In chronic diabetes, kidney weight is increased and serum BUN and creatinine levels are elevated due to swelling, inflammation, and necrotic processes. BUN and creatinine tests measure the amount of urea nitrogen and non-protein nitrogenous product of muscle in the blood, respectively, that gives an estimate of how well the kidneys are functioning (glomerular filtration). We found that ZG02-ME treatment significantly decreased serum BUN and creatinine levels in *db*/*db* mice compared with the vehicle control group. It is also well known that free radicals contribute to the etiology of diabetes and alter antioxidant defense. Hyperglycemia-generated free radicals are formed by glucose auto-oxidation in diabetes. Glucose auto-oxidation has been linked to non-enzymatic glycosylation, and glycosylated proteins provide a source of free radicals [47,48]. Thus, oxidative stress plays an important role in the etiology of diabetic complications. Elevated level of lipid peroxidation in various organs, including liver and kidney, can increase generation of harmful reactive oxygen species and causes organ damage and dysfunction [49,50]. We observed that diabetic mice had increased lipid peroxidation (TBARS) levels that were decreased by ZG02-ME treatment both in liver and kidney. Taken together, these findings suggest that ZG02-ME has an inhibitory activity against diabetic hepatopathy and nephropathy in type 2 diabetes.

## 5. Conclusions

In conclusion, we identified ZG02-ME as an anti-diabetic compound that effectively ameliorated glucose and lipid metabolism, and improved metabolic hormones in a type 2 diabetic animal model. Furthermore, ZG02-ME treatment improved liver and kidney functions in *db*/*db* mice. Our findings clearly demonstrated effective therapeutic properties of ZG02-ME in *db*/*db* mice, suggesting ZG02-ME is a promising bioactive agent for diabetes treatment.
